# Cannabidiol Treatment for Adult Patients with Drug‐Resistant Epilepsies: A Real‐World Study in a Tertiary Center

**DOI:** 10.1002/brb3.70122

**Published:** 2024-11-05

**Authors:** Quentin Calonge, Aurore Besnard, Laurent Bailly, Maria Damiano, Phintip Pichit, Sophie Dupont, Isabelle Gourfinkel‐An, Vincent Navarro

**Affiliations:** ^1^ AP‐HP, Epilepsy Unit, Department of Neurology, Reference Center of Rare Epilepsies ERN‐EpiCare, Pitié‐Salpêtrière Hospital Paris France; ^2^ Paris Brain Institute, ICM, Inserm, CNRS Sorbonne Université Paris France; ^3^ AP‐HP, EEG Unit, Department of Neurophysiology Pitié‐Salpêtrière Hospital Paris France; ^4^ Rehabilitation Unit, AP‐HP Pitié‐Salpêtrière Hospital Paris France

**Keywords:** cannabidiol, drug‐resistant epilepsy, safety, therapeutics

## Abstract

**Background and purpose:**

Around 30% of patients with epilepsy show drug‐resistant epilepsy (DRE). While cannabidiol has demonstrated efficacy as an adjunctive treatment in Dravet syndrome (DS), Lennox–Gastaut Syndrome (LGS), and epilepsy related to tuberous sclerosis complex (TSC), its more global effectiveness in adult patients with DRE apart from these three specific contexts needs to be clarified.

**Methods:**

We conducted a retrospective study at the epilepsy unit of Pitié Salpêtrière Hospital. Patients initiating pharmaceutical cannabidiol treatment and followed for at least 1 year were included. Patients were categorized into “authorized” (LGS, DS, or TSC) and “off‐label” groups. Cannabidiol effectiveness and tolerance were compared between groups, and characteristics of responders (patients with >50% reduction in seizure frequency) in the off‐label group were examined.

**Results:**

Ninety‐one patients, followed by a median duration of 24 months, were included. A total of 35.2% of the patients were in the authorized group. No significant differences were observed in responder rates between groups (31.3% vs. 35.6%, *p* = 0.85) and retention rates at 1 year (75.0% vs. 74.6%, *p* = 0.97). Sleepiness was more commonly reported in the authorized group (50.0% vs. 22.0%, *p* = 0.01), with no other significant differences. Among off‐label patients (*n* = 59), clobazam co‐prescription was more prevalent in responders (71.4% vs. 28.9%, *p* = 0.002).

**Conclusion:**

Our findings suggest that cannabidiol may benefit all adult patients with DRE, particularly those already receiving clobazam. Randomized controlled trials are warranted in off‐label patients to validate these observational findings.

## Graphical abstract

We extracted all adult patients treated at our tertiary center with cannabidiol for pharmacoresistant epilepsy, with a follow‐up of at least 1 year, and evaluated the efficacy and retention based on the indication. We found similar efficacy and retention rates in patients with Lennox–Gastaut syndrome, Dravet syndrome, and tuberous sclerosis complex compared to other forms of pharmacoresistant epilepsy.

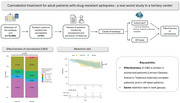

## 1 Introduction

Epilepsy is one of the most prevalent neurological disorders, with an estimated prevalence of 7.6 per 1000 persons worldwide (Fiest et al. 2017). Approximately one‐third of patients exhibit drug‐resistant epilepsy (DRE) which is associated with heightened morbidity and mortality (Perucca et al. 2023). Cannabidiol has demonstrated efficacy in randomized controlled trials in three indications: Lennox–Gastaut syndrome (LGS), Dravet syndrome (DS), and epilepsy related to tuberous sclerosis complex (TSC) (Devinsky et al. 2017; Thiele et al. 2021, 2018). In France, authorization for its use was granted in 2018 for LGS, DS, and TSC in patients aged 2 years and more. However, evidence suggesting the efficacy of cannabidiol in pharmacoresistant epilepsies of various etiologies has emerged from several small case series (Lattanzi et al. 2021) and subsequent real‐world studies, predominantly involving children or adults with LGS, DS, or TSC (Kühne et al. 2023; Perriguey et al. 2024). Our study reports the efficacy and tolerability of cannabidiol in a cohort of adults with DRE of diverse causes.

## 2 Materials and Methods

A retrospective review was conducted on all patients followed at the epilepsy unit at Pitié Salpêtrière Hospital from January 2018 up to April 2024, encompassing 10,450 patients. Patients were identified if the terms “Cannabidiol,” “CBD,” or “Epidyolex” appeared in any of their medical records. Inclusion criteria comprised two conditions: (i) initiation of a pharmaceutical formulation of highly purified cannabidiol treatment (Epidyolex^R^) by a physician from the Pitié Salpêtrière epilepsy unit for a DRE and (ii) a minimum duration of 1 year between the initiation of treatment and the last consultation/hospitalization in the epilepsy unit. Patients who died within 1 year after treatment initiation were also included.

This study was conducted according to French legislation and authorized by the French data protection authority CNIL (No. 2211991). According to the Declaration of Helsinki, patients were informed that their anonymized data would be used in this study.

For each patient, we extracted age at the initiation of cannabidiol, sex, epilepsy etiology, presence of epileptic encephalopathy, genetic abnormality or brain lesion, number of antiseizure medications at the time of cannabidiol introduction, co‐prescription of clobazam, presence of an active vagus nerve stimulator (VNS), maximum daily dose of cannabidiol relative to weight (mg/kg/day). The effectiveness of the treatment for all seizure types was documented by the clinician at the patient's last follow‐up and was categorized as “no effectiveness on frequency,” “< 25% reduction,” “25%–50% reduction,” “50%–75% reduction,” or “more than 75% reduction.” Responder patients were defined as those with a reduction in seizure frequency of more than 50%. Additionally, other efficacy measures such as reduction in seizure intensity or duration and cognitive‐behavioral improvement were extracted, as all adverse events reported by physicians.

The primary objective was to compare the effectiveness and safety of cannabidiol between two subgroups of patients: the “authorized” group composed of patients for whom the treatment was administered in approved contexts (LGS, DS, or TSC) and the “off‐label” group composed of the other patients receiving cannabidiol treatment off‐label.

The secondary objective was to compare responders and non‐responders in the “off‐label” population to delineate possible features of responders.

Categorical variables were summarized as counts and percentages, while numerical variables were presented as medians and inter‐quartile ranges. Proportions were compared using either a chi‐square or Fisher's exact test, and continuous variables using a either *t*‐test or Wilcoxon test, based on the normality assumption and sample size. Retention curves were constructed using the Kaplan–Meier method, and comparisons between authorized and off‐label patients were conducted using the log‐rank test.

A threshold of 0.05 was taken as significant, and all analyses were performed using R studio version 4.1.3.

## 3 Results

Extraction was conducted on March 12, 2024, resulting in the identification of 398 patients. Among them, 244 had never received cannabidiol, 23 patients started their treatment outside the Pitié‐Salpêtrière epilepsy unit, and 2 patients had indications unrelated to epilepsy. Furthermore, eight patients were prescribed therapeutic cannabis instead of cannabidiol, and medical records for 14 lacked sufficient information. Lastly, the follow‐up duration from the start of treatment was less than 1 year for 17 patients. A total of 91 patients were included in the analysis, comprising 32 patients with one of the authorized indications (21 LGS, 8 DS, 3 TSC) and 59 patients with various causes of DRE (Table 1) with a median duration of follow‐up of 24 months (Interquartile range, [IQR]: 19–30).

**TABLE 1:** Causes of epilepsy among the off‐label group.

Cause	Number of patients (total: 59)
Genetics/chromosomal abnormality	24
STXBP1	2
CACNA1E	1
NTRK2	1
SLCGA1	1
ARX	1
FOXG1	1
PPP3CA	1
KCNT1	1
SYNGAP1	1
SLC6A8	1
ATP1A3	1
CHD2	1
Ring chromosome 20	2
1p36 deletion	2
15q‐15q tetrasomy	2
16q24 deletion	1
17q12 deletion	1
Chromosome 18 short arm anomaly	1
Trisomy 21	1
Rett syndrome	1
Cerebral malformations	7
Cortical dysplasia	3
Pachygyria	2
Double cortex	2
Other	12
Perinatal anoxia	4
Post‐infectious encephalitis	3
FIRES	2
Lance–Adams syndrome	2
Hemiplegia/convulsion	1
Unknown	16

### 3.1 Comparison of the Two Groups

In the off‐label group, there was a higher proportion of women compared to the authorized group, although this difference did not reach statistical significance (54.2% vs. 31.3% in the authorized group, *p* = 0.06). No significant differences were observed between the two groups regarding age, number of treatments, co‐prescription of clobazam, presence of activated VNS, treatment duration, and maximum daily dose/weight (Table 2).

**TABLE 2:** characteristics of patients, efficacy, and tolerance according to the indication of cannabidiol.

Clinical variable	Authorized *n* = 32	Off‐label *n* = 59	*p* value
Sex (female)	10 (31.3%)	32 (54.2%)	0.06
Age (mean and standard deviation)	29.5 (7.57)	31.3 (10.1)	0.36
Number of concomitant antiseizure medications (median and range)	3 (0–7)	3 (1–6)	0.43
Active vagus nerve stimulator	9 (28.1%)	21 (35.6%)	0.70
Clobazam co‐prescription	16 (51.6%)	26 (44.1%)	0.75
Treatment duration of CBD (months) (median and interquartile range)	27 (20–34)	24 (18.5–27.5)	0.11
Maximal treatment dose (mg/kg/day) (median and interquartile range)	10.7 (8.63–12.4)	11.1 (8.92–14.1)	0.35
Effectiveness			
>50% reduction of seizure frequency	10 (31.3%)	21 (35.6%)	0.85
Less “intense” seizures	3 (9.4%)	10 (16.9%)	0.53
Shorter seizures	3 (9.4%)	5 (8.5%)	1
Cognitive‐behavioral improvement	11 (34.4%)	17 (28.8%)	0.76
Side‐effects			
All	20 (62.5%)	24 (40.7%)	0.08
Sleepiness	16 (50.0%)	13 (22.0%)	0.01*
Behavioral disorders	2 (6.3%)	5 (8.5%)	1
Increased seizures	2 (6.3%)	5 (8.5%)	1
Liver balance disturbance^a^	2 (6.3%)	1 (1.7%)	0.28
Other side effects	1 (3.1%)	4 (6.8%)	0.65

^a^ “Liver balance disturbances” refers to an elevation in liver transaminase levels or bilirubin levels. * *p* < 0.05.

**FIGURE 1:**
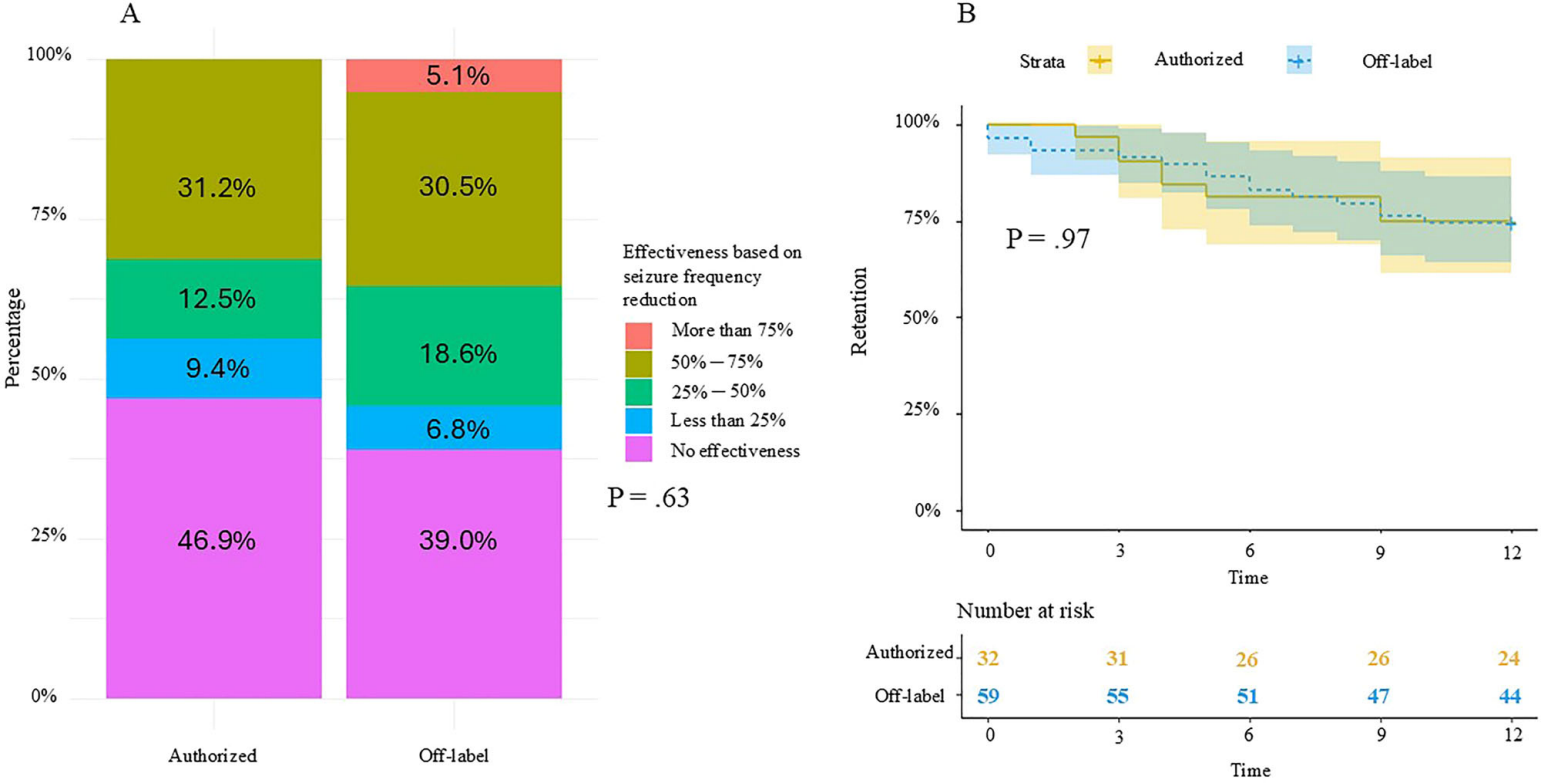
Comparison of effectiveness, evaluated on the seizure frequency reductions between the authorized and off‐label groups, and retention at 1 year according to group (authorized or off‐label).

The responder rate was 31.3% in the authorized group and 35.6% in the off‐label group, with no significant difference between the two groups (*p* = 0.85). Additionally, the distribution of response levels did not differ between the two groups (Figure 1A, p = 0.63). There was no difference between the two groups in reported effectiveness on seizure intensity (9.4% vs. 16.9%, *p* = 0.43), duration, and cognitive‐behavioral improvement (34.4% vs. 28.8%, *p* = 0.76).

Retention rates at 1 year were 75.0% in the authorized group and 74.6% in the off‐label group, with no significant difference (*p* = 0.97, Figure 1b). Somnolence was the most frequently reported adverse event with a higher prevalence in the authorized group compared to the off‐label group (50.0% vs. 22.0%, *p* = 0.01), while other adverse events did not differ between the two groups (Table 1). Among the 23 patients who discontinued treatment within 1 year, 13 (56.5%) discontinued due to lack of efficacy, 4 (17.4%) due to worsening of seizures, and 6 (26.1%) due to tolerance issues (two liver disturbances, two behavioral disorders, one vomiting, and one disabling cough). Among patients continuing treatment beyond 1 year, there were 12 discontinuations: eight (66.7%) due to lack of efficacy, one for death, one for behavioral problems, one for worsening seizures, and one for tolerance.

### 3.2 Comparison Between Responders and Non‐Responders in the off‐Label Group

In the off‐label group, the only significant difference between responders and non‐responders was the more frequent co‐prescription of clobazam among responders (71.4% vs. 28.9%, *p* = 0.002, Table S1) which was also the case for all patients (64.5% of responders vs. 36.7%, *p* = 0.02).

## 4 Discussion

Our study described the real‐world effectiveness of cannabidiol treatment in adult patients with DRE of various etiologies, in a large cohort followed for at least 1 year. We found no significant difference in effectiveness among off‐label patients (responder rate of 35.6%) compared to authorized patients (31.3%). This later responder rate is relatively similar than those reported in randomized trials for LGS for the dose of 10/mg/kg/day (36%) (Devinsky et al. 2018) and remains comparable to those reported in real‐world studies in adult patients (Kühne et al. 2023; Perriguey et al. 2024). In these later studies, the maximum doses of CBD reached in adults were comparable to those in our study, at a median of around 10 mg/kg/day which is lower than the maximum possible dose set in France at 20 mg/kg/day. In addition, the overall percentage of patients showing a reduction of seizures frequency (including those with less than 25%) was 58.2%, suggesting that the treatment was able to bring benefit to more than 50% of patients. Two recent real‐world studies involving children and adults suffering from DRE other than LGS, DS, and TSC found high responder rates (50% and 68.8%, respectively) albeit without comparison to patients suffering from LGS, DS, or TSC (Ferrera et al. 2023; Espinosa‐Jovel et al. 2023). Even if the exact mechanism of action of cannabidiol in human DRE is unknown, several targets have been proposed to explain the antiseizure properties of CBD including functional antagonism of GRP55 receptor, inhibition of adenosine reuptake, and desensitization of TRPV1 receptors (Gray and Whalley 2020). None of those targets are specific to LGS, DS, or TSC, and our study suggests that the effectiveness of cannabidiol does not differ between adult patients with one of those syndromes or other forms of DRE. The most frequently reported adverse event in our study was sleepiness, consistent with findings from other studies, although it rarely led to treatment discontinuation (Georgieva et al. 2023). However, the percentage of patients reporting somnolence in the authorized group (50%) was higher than in other real‐world studies (Devinsky et al. 2018; Ferrera et al. 2023; Kühne et al. 2023; Perriguey et al. 2024). It is noteworthy that “fatigue” was reported in 36% of DS patients in a randomized trial (Devinsky et al. 2017), suggesting its potential frequency and partly explaining these findings in our study. Here, we considered any mention of unusual fatigue as a possible adverse effect of cannabidiol.

The only difference found between responders and non‐responders in the off‐label group was the presence of a co‐prescription of clobazam, which was more prevalent among responders. It has been reported that the potential synergistic effect between cannabidiol and clobazam may be linked to inhibition of cannabidiol‐metabolizing cytochromes (Anderson et al. 2019). A recent meta‐analysis involving 714 subjects across four studies found a responder rate of 52.9% in patients receiving cannabidiol with clobazam, whereas it was only 29.1% in patients receiving cannabidiol without clobazam (Lattanzi et al. 2020). This greater effectiveness in co‐exposed patients has been reported in other real‐life studies (Perriguey et al. 2024). However, our study is, to our knowledge, the first to identify this possible synergistic effect between cannabidiol and clobazam in off‐label patients.

Our study suffers from the weaknesses of retrospective studies, in particular biases in data collection may arise from reliance on information reported by clinicians in their reports. Some information, such as the precise number of seizures at baseline or after CBD introduction, was not consistently available, and caution must be exercised in inferring causal associations from our findings.

Nevertheless, our study suggests that cannabidiol could be proposed in all adult patients with drug‐resistant epilepsy, particularly if they are already being treated with clobazam, although these results remain to be confirmed in a randomized controlled trial.

## Author Contributions

**Quentin Calonge**: conceptualization, data curation, formal analysis, methodology, writing–original draft, writing–review and editing. **Aurore Besnard**: conceptualization, data curation, writing–review and editing. **Laurent Bailly**: conceptualization, resources, writing–review and editing. **Maria Damiano**: writing–review and editing, resources. **Phintip Pichit**: resources, writing–review and editing. **Sophie Dupont**: resources, writing–review and editing. **Isabelle Gourfinkel‐An**: conceptualization, resources, writing–review and editing. **Vincent Navarro**: conceptualization, funding acquisition, resources, writing–original draft, writing–review and editing.

## Conflicts of Interest

P.P. received honorarium from Livanova for conferences. S.D. received honorarium from Esai, Bial, Angelini, Jazz Pharma, and Sanofi for Advisory boards and conferences. L.B. received honorarium from Jazz Pharma, UCB pharma and Biocodex for Advisory boards. I.G.‐A. received honorarium from Jazz Pharma and UCB Pharma for Advisory boards and conferences. V.N. received honorarium from UCB pharma, EISAI, Jazz Pharma, Angellini, Neuraxpharm France for Advisory boards and conferences. All other authors declare no other conflicts of interest.

### Peer Review

The peer review history for this article is available at https://publons.com/publon/10.1002/brb3.70122.

## Supporting information




**Supplementary Table 1**: difference between responders (reduction in seizure frequency of more than 50%) and non‐responders in the off‐label group

## Data Availability

The data that support the findings of this study are available on request from the corresponding author. The data are not publicly available due to privacy or ethical restrictions.
