# Supplementary material for: Cannabidiol Treatment for Adult Patients with Drug‐Resistant Epilepsies: A Real‐World Study in a Tertiary Center
Source: Brain Behav. 2024 Nov 5;14(11):e70122. doi: 10.1002/brb3.70122 (PMC11538088; doi:10.1002/brb3.70122)
Supplement: Supplementary file 1 — Supplementary Table 1: difference between responders (reduction in seizure frequency of more than 50%) and non‐responders in the off‐label group [file BRB3-14-e70122-s001.docx]

**Supplementary table 1**: difference between responders (reduction in seizure frequency of more than 50%) and non-responders in the off-label group

| Clinical variable | **Responders**  **n = 21** | **Non-responders**  **n = 38** | p-value |
| --- | --- | --- | --- |
| Sex (Female) | 10 (47.6%) | 22 (57.9%) | 0.63 |
| Age | 30.9 (6.22) | 31.4 (11.7) | 0.33 |
| Number of concomitant antiseizure medications (median and range) | 4 (1 – 6) | 3 (0 – 6) | 0.06 |
| Active vagus nerve stimulator | 6 (28.6%) | 15 (39.5%) | 0.57 |
| Clobazam co-prescription | 15 (71.4%) | 11 (28.9%) | 0.002* |
| Treatment duration of CBD (months) | 23 (19 – 27) | 24 (18.2 – 27.8) | 1 |
| Maximal treatment dose (mg/kg/d) | 11.3 (9.41 – 12.2) | 11.0 (8.78 – 15) | 0.96 |
| Epileptic encephalopathy | 14 (66.7%) | 28 (73.7%) | 0.57 |
| Genetic abnormality | 10 (47.6%) | 16 (42.1%) | 0.79 |
| MRI lesion | 8 (38.1%) | 15 (39.5%) | 1 |
